# Staircase array of inclined refractive multi-lenses for large field of view pixel super-resolution scanning transmission hard X-ray microscopy

**DOI:** 10.1107/S1600577521001521

**Published:** 2021-03-12

**Authors:** Talgat Mamyrbayev, Katsumasa Ikematsu, Hidekazu Takano, Yanlin Wu, Kenji Kimura, Patrick Doll, Arndt Last, Atsushi Momose, Pascal Meyer

**Affiliations:** a Institute of Microstructure Technology, Karlsruhe Institute of Technology, Hermann-von-Helmholtz-Platz 1, 76344 Eggenstein-Leopoldshafen, Baden-Württemberg, Germany; b Institute of Multidisciplinary Research for Advanced Materials, Tohoku University, 2-1-1 Katahira, Aoba-ku, Sendai, Miyagi 980-8577, Japan

**Keywords:** inclined refractive X-ray multi-lens array, pixel super-resolution, scanning transmission hard X-ray microscopy, deep X-ray lithography and electroplating

## Abstract

Owing to the development of X-ray focusing optics during the past decades, synchrotron-based X-ray microscopy techniques allow the study of specimens with unprecedented spatial resolution, down to 10 nm, using soft and medium X-ray photon energies, though at the expense of the field of view (FOV). One of the approaches to increase the FOV to square millimetres is raster-scanning of the specimen using a single nanoprobe; however, this results in a long data acquisition time. This work employs an array of inclined biconcave parabolic refractive multi-lenses (RMLs), fabricated by deep X-ray lithography and electroplating to generate a large number of long X-ray foci. Since the FOV is limited by the pattern height if a single RML is used by impinging X-rays parallel to the substrate, many RMLs at regular intervals in the orthogonal direction were fabricated by tilted exposure. By inclining the substrate correspondingly to the tilted exposure, 378000 X-ray line foci were generated with a length in the centimetre range and constant intervals in the sub-micrometre range. The capability of this new X-ray focusing device was first confirmed using ray-tracing simulations and then using synchrotron radiation at BL20B2 of SPring-8, Japan. Taking account of the fact that the refractive lens is effective for focusing high-energy X-rays, the experiment was performed with 35 keV X-rays. Next, by scanning a specimen through the line foci, this device was used to perform large FOV pixel super-resolution scanning transmission hard X-ray microscopy (PSR-STHXM) with a 780 ± 40 nm spatial resolution within an FOV of 1.64 cm × 1.64 cm (limited by the detector area) and a total scanning time of 4 min. Biomedical implant abutments fabricated via selective laser melting using Ti–6Al–4V medical alloy were measured by PSR-STHXM, suggesting its unique potential for studying extended and thick specimens. Although the super-resolution function was realized in one dimension in this study, it can be expanded to two dimensions by aligning a pair of presented devices orthogonally.

## Abstract 2

A new X-ray focusing device generates hundreds of thousands of line foci, periodically spaced in the sub-micrometre range, with centimetre length. It enables to achieve large FOV pixel super-resolution scanning transmission hard X-ray microscopy.

## 1. Introduction   

X-ray focusing optics are key elements for increasing the performance of X-ray beams at synchrotron sources. The first demonstration of the possibility of X-ray focusing in X-ray microscopy by utilizing Kirkpatrick–Baez (KB) mirrors (Kirkpatrick & Baez, 1948 ▸) has gained the rapid development of various types of X-ray optics based on reflection (Mimura *et al.*, 2008 ▸; Yumoto *et al.*, 2013 ▸; Matsuyama *et al.*, 2017 ▸, 2015 ▸), diffraction (Vila-Comamala *et al.*, 2011 ▸; Mohacsi *et al.*, 2017 ▸; Bajt *et al.*, 2018 ▸) and refraction phenomena (Snigirev *et al.*, 1996 ▸; Patommel *et al.*, 2017 ▸; Schroer & Lengeler, 2005 ▸). The development of the focusing system was mainly targeted at the achievement of the smallest X-ray focus in the nanometre range. Nowadays, the sub-10 nm-sized X-ray probe can be reached at high-brilliance synchrotron sources using photon energies below 20 keV (Mimura *et al.*, 2010 ▸; Yamauchi *et al.*, 2011 ▸; Döring *et al.*, 2013 ▸). As a consequence, owing to the high penetration depth of X-rays, X-ray microscopy techniques are widely used for non-destructive testing and visualization of the inner structure of specimens, providing extremely high spatial resolution equal to the X-ray nanoprobe size, though at the expense of the FOV (Sakdinawat & Attwood, 2010 ▸). Scanning transmission X-ray microscopy (STXM) allows enlargement of the FOV up to several hundred micrometres by raster-scanning of a specimen through a single X-ray nanoprobe. At each specimen position, X-ray intensity variation caused by the absorption of the specimen is registered by the detector, and the two-dimensional X-ray image is reconstructed in a pixel-by-pixel manner. However, X-ray microscopy of centimetre-sized specimens with sub-micrometre spatial resolution will need an extremely long data acquisition time.

Recently, the pixel super-resolution scanning transmission hard X-ray microscopy (PSR-STHXM) technique (Mamyrbayev *et al.*, 2019 ▸, 2020 ▸) was demonstrated, which allows an increase of the FOV and drastically reduces the scanning time owing to sub-pixel specimen scanning through a large number of X-ray probes created by an array of biconcave parabolic refractive lenses. The specimen is placed in the focal distance and illuminated by the X-ray foci. At each specimen position, low-resolution (LR) images are recorded by the detector. The number of LR images is defined by the distance between neighboring foci and the scanning step size. The term ‘pixel super-resolution’ (PSR) refers to an image-processing (reconstruction) approach for resolution enhancement. A high-resolution image is obtained from a set of sub-pixel shifted LR images by stacking LR pixel values via PSR reconstruction. It is possible to reconstruct images with a spatial resolution corresponding to the X-ray (micro-) nanoprobe size, even if the pixel size of the imaging detector employed for data acquisition is much larger. As a result, an FOV of 1.67 mm × 1.67 mm (restricted by the geometry of the lens array) at 34 keV with a scanning time of several minutes was demonstrated (Mamyrbayev *et al.*, 2020 ▸).

However, the FOV is limited by the height of the lens pattern fabricated by the deep X-ray lithography technique, which is in the millimetre range at the maximum. It should be mentioned that other fabrication techniques of X-ray lens arrays such as silicon etching (Snigirev *et al.*, 2014 ▸) and polymer additive manufacturing (Mikhaylov *et al.*, 2020 ▸) also cannot provide the structures for a centimetre-order FOV. The idea is based on fabricating many inclined biconcave parabolic refractive multi-lenses (RMLs) at regular intervals on a substrate. By tilting the substrate against X-rays, the RMLs generate X-ray line foci in the centimetre range, resulting in a large FOV. An analogous approach for increasing the structure height of the diffractive optical elements in a tilted geometry was demonstrated by Karvinen *et al.* (2014 ▸) and Lebugle *et al.* (2017 ▸).

**Figure 1:**
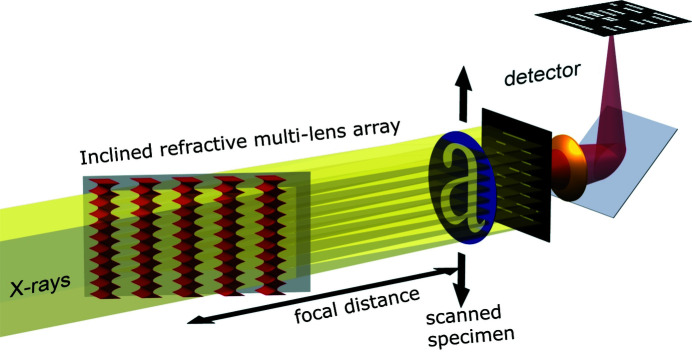
Concept of large FOV PSR-STHXM based on the inclined RML array. Under monochromatic plane-wave X-ray illumination, the array of RMLs generates X-ray line foci with a period equal to that of the multi-lens. The specimen is placed at the focal position and scanned through the X-ray line foci with step sizes slightly below the size of the line focus (FWHM). At each specimen step, an image [‘low-resolution (LR) image’ hereafter] is recorded. A high-resolution image is reconstructed from a set of LR images by the pixel super-resolution reconstruction algorithm.

The new optical element has 189 RMLs, and each RML consists of 2000 lens elements; that is, 378 000 identical single nickel lenses were fabricated via tilted exposure deep X-ray lithography and electroplating. PSR-STHXM experiments, whose setup is shown in Fig. 1 ▸, with a 780 ± 40 nm spatial resolution, a 1.64 cm × 1.64 cm FOV and a 4 min scanning time at 35 keV, will be demonstrated with the RML array.

## 2. Principle and design of inclined RML array   

**Figure 2:**
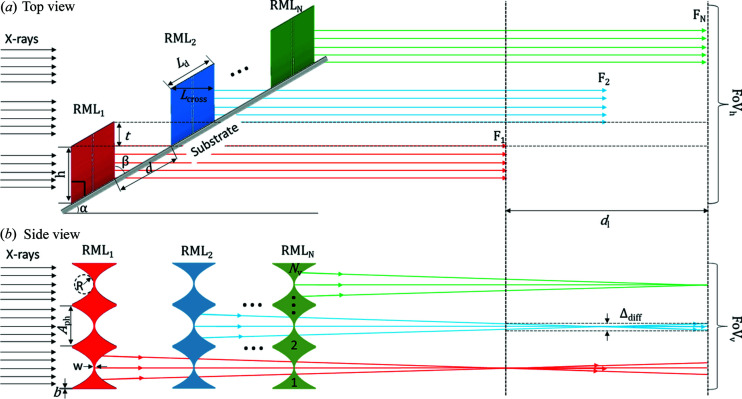
Concept and design parameters of the array of inclined RMLs for large FOV PSR-STHXM. (*a*) Top view. (*b*) Side view [*d* – distance between RMLs; Δ_diff_ – diffraction-limited spot size; FOV_h_ and FOV_v_ – horizontal and vertical FOVs in the detector plane, respectively; *F_i_* (*i* = 1, 2, …, *N*) – the distances of focal positions measured from RML_1_; *w* – web distance; *A*_ph_ – physical aperture; *R* – radius of curvature; *b* – distance between neighboring parabolas; α – tilt angle of the substrate; β – inclination angle of the RML against the substrate; *L*_d_ – length of the lens; *L*_cross_ – lens length along with X-rays; *h* – height of the structures when the substrate is tilted; *t* – transition zone where two RMLs contribute to form line foci; *d*_l_ – depth of field; *N*_V_ – number of lenses in each RML].

The working principle of increasing the length of X-ray line foci by one-dimensional RMLs is shown in Fig. 2 ▸. It follows the strategy to utilize an array of inclined RMLs with regular intervals *d* and uniform height *h*. Furthermore, the inclination angle β of all RMLs is identical on the substrate. Consequently, by tilting the substrate to the angle α = 90° − β relative to the beam, the RMLs are positioned perpendicularly to the optical axis and are placed over the whole FOV_h_ within a staircase-like arrangement. Moreover, in the transition area *t* (*t* = *h* − *d*cosβ) the top part of one RML and the bottom part of the next RML contribute to the focusing of the incoming X-ray beam. Thus, under monochromatic plane wave X-ray illumination, the RMLs produce X-ray line foci with a length in the centimetre range, which is defined by the number of RMLs [Fig. 2 ▸(*a*)]. However, in the perpendicular direction in Fig. 2 ▸(*b*) (specimen scanning direction), the FOV_v_ is equivalent to the number of lenses in a single RML.

The inclined RML array was designed especially for application in PSR-STHXM. The aperture of the lens in an RML can be equal to the pixel size or a multiple of it. Each lens has a biconcave parabolic profile as the refractive index for X-rays in matter is <1 [Fig. 2 ▸(*b*)] (Lengeler *et al.*, 1999*a* ▸). The depth of parabola *l* of the lens is defined as
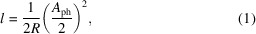
where *R* is the radius of curvature in the apex of the parabola of the refracting surface. Then, *L*_d_ [see Fig. 2 ▸(*a*)] should be
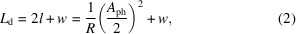
taking account of the web distance *w*. Correspondingly, when the substrate is tilted at the angle α = 90° − β, the length of the X-ray path through an RML, *L*_cross_, is
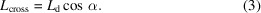
The focal distance *f* of each RML is calculated as (Lengeler *et al.*, 2002 ▸)
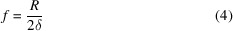
where δ is the refractive index decrement from the unity of the lens material. For nickel at 35 keV, δ = 1.44 × 10^−6^.

The width of the X-ray line focus (FWHM) is given by the diffraction-limited spot size; that is (Kohn *et al.*, 2003 ▸)
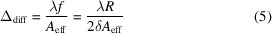
where λ is the wavelength and *A*_eff_ is the effective aperture of the single-lens, which is related to the physical aperture 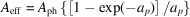, *a_p_* includes the X-ray attenuation in the lens material and surface roughness (Lengeler *et al.*, 1999*a* ▸,*b* ▸).

The depth of focus (DOF) of the single lens has to be large enough to obtain sub-micrometre focal width (FWHM) within the depth of field *d_l_* (>*F*_N_ − *F*_1_, see Fig. 2 ▸) and is calculated by (Lengeler *et al.*, 1999*a* ▸)
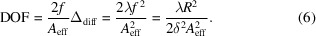
The FOV in the vertical direction (FOV_v_), which corresponds to the specimen scanning direction, and FOV_h_ in the horizontal direction are given by
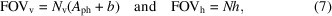
where *N*_V_ is the number of lenses in single RML, *b* is the distance between neighboring parabolas and *N* is the number of RMLs.

Since the RMLs consist of individual biconcave parabolic lenses, the requirement of monochromaticity is the same as for conventional refractive X-ray lenses which are chromatic. Increasing the value of monochromaticity (Δ*E*/*E*) leads to chromatic aberrations. The relationship between the focal distance (*f*) and monochromaticity is Δ*f*/*f* = 2Δ*E*/*E*. The typical value is Δ*E*/*E* = 10^−4^ for monochromatic light.

## 3. Ray-tracing simulations of the influence of the height mismatch and angular misalignment on the focusing performance in the transition area   

**Figure 3:**
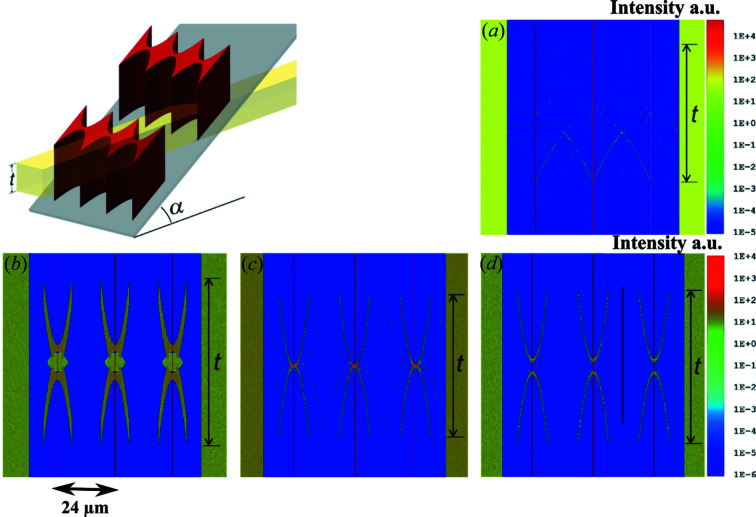
Ray-tracing simulation of the intensity distribution in the transition area *t* of the inclined RML array (see Fig. 2 ▸) made of nickel at an X-ray energy of 35 keV. The distance between two neighbor line foci is 24 µm, which is equal to the aperture of the lenses. (*a*) Perfect RMLs provide three parallel focus lines appearing in red with nearly no background feature. (*b*) The background feature is prominent when neighbor RMLs have a 2 µm height difference. Background features when the substrate is not perfectly inclined to α = 30°, but to (*c*) α = 29.8° and (*d*) α = 30.2°.

Ray-tracing simulations have been performed to study the influence of alignment errors and manufacturing tolerances on the optical performance of the optics in the transition area *t*, using the commercial software *Zemax* (Zemax, LLC, Delaware limited liability company, USA). The ray-tracing simulations were performed, taking into account the inclined RMLs concerning the substrate and substrate tilting, as shown in Fig. 3 ▸. An area of three lenses in a pair of RMLs has been used for simulation, so the resulting images show three parallel line foci [Fig. 3 ▸(*a*)]. The color at the left and right borders of the images indicate the intensity level of the incoming ray. Note that many more rays were used to perform the simulation for the ideal RMLs to at least see the faint background; 80.1 million rays were used for the simulation results depicted in Figs. 3 ▸(*a*), and 20 million for the simulation results depicted in Figs. 3 ▸(*b*), 3(*c*) and 3(*d*).

Rays which pass through two lens elements in the transition area *t* shown in Fig. 2 ▸(*a*) are thus focused just as well as rays which pass through only one lens element. The background level around the line foci in the transition area is in the range of 10^−6^ of the focused intensity.

The height of the RMLs should be constant over the whole area, but the top side of the nickel height may have a distribution after the electroplating process. To simulate the effects of such height deviation, a height difference of 2 µm between neighboring RMLs was introduced. As presented in Fig. 3 ▸(*b*), in this case, a background feature reaching tenfold intensity of the incident beam is produced. The effect of the error in substrate tilting is also simulated in Figs. 3 ▸(*c*) and 3(*d*) where α = 29.8° and 30.2°, respectively. The focusing performance would not be degraded seriously if the height of the RMLs is made constant, for instance, by a polishing procedure after the electroplating and by inclining the substrate precisely.

## 4. Inclined RML array fabrication   

**Figure 4:**
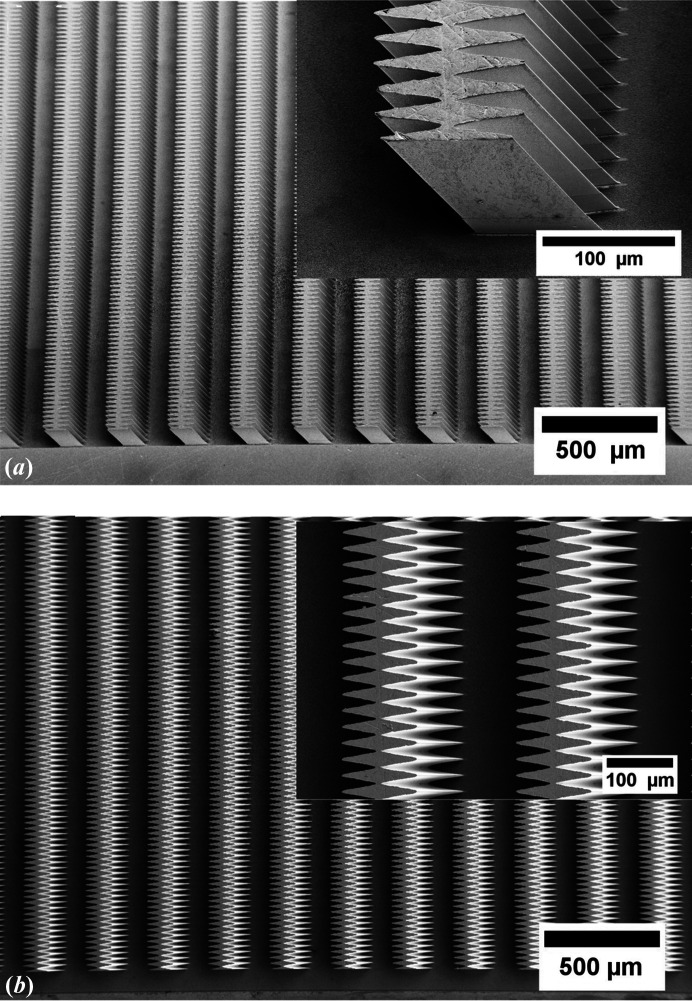
SEM images of the inclined RML array: (*a*) bird’s eye view and (*b*) top view. The inclination angle of the nickel RMLs to the substrate is 60°. (*b*) All structures are identical with the same parabolic profiles and are well distanced according to the design values.

The inclined RML array was fabricated via tilted exposure deep X-ray lithography and electroplating (Nazmov *et al.*, 2011 ▸; Meyer & Schulz, 2015 ▸). An X-ray-positive photoresist layer was glued onto the silicon substrate with a thickness of 525 µm covered with a 2.5 µm conductive seed layer (titanium with an oxidized surface). Oxidation of the titanium surface increased the surface roughness and provided excellent adhesion of the photoresist and electroplated metal structures. Polymethyl­methacrylate (PMMA) was used as the photoresist with a thickness of 232 µm. An X-ray absorption working mask pattern was transferred onto the photoresist layer using highly collimated X-rays from KARA synchrotron beamline LITHO II. After exposure and development of the irradiated parts in a developer solution, the pattern needed for the RML array was obtained. The nickel structures were created by an electroplating process (Nazmov *et al.*, 2005 ▸; Snigirev *et al.*, 2004 ▸). The precise height of the inclined RML array is a crucial parameter. Therefore, after the electroplating process, the structures were polished without stripping of the PMMA resist, providing additional stability and preventing structures from collapsing during the polishing process. To achieve a uniform height, the surface of the inclined RML array was ground using different silicon carbide (SiC) grinding papers (P400-P2500; Struers GmbH, Germany). Then, when a structure height of about 118 µm was achieved, the inclined RML array was rinsed in deionized water and dried with compressed nitro­gen. After that, the PMMA resist was exposed at KARA/LITHO II and removed by a stripping process. Scanning electron microscopy (SEM) images of the fabricated inclined RML array are shown in Fig. 4 ▸. The design and fabricated parameters of the inclined RML array shown in Fig. 4 ▸ are listed in Table 1 ▸.

The measurements of the height and the inclination angle β of the RMLs were performed using an optical three-dimensional surface profiler (Contour GT, Brucker, USA) working in vertical scanning interferometry mode when the substrate was in the horizontal position (α = 0°); the RMLs parameters (*d*, *w*, *A*_ph_, *L*_d_, *R*) were measured by SEM (ZEISS Supra 60VP). The values when the substrate with inclined RMLs is tilted at an angle α = 30° were calculated from the measured ones and compared with the designed values.

## 5. Evaluation of the inclined RML array by synchrotron radiation   

**Figure 5:**
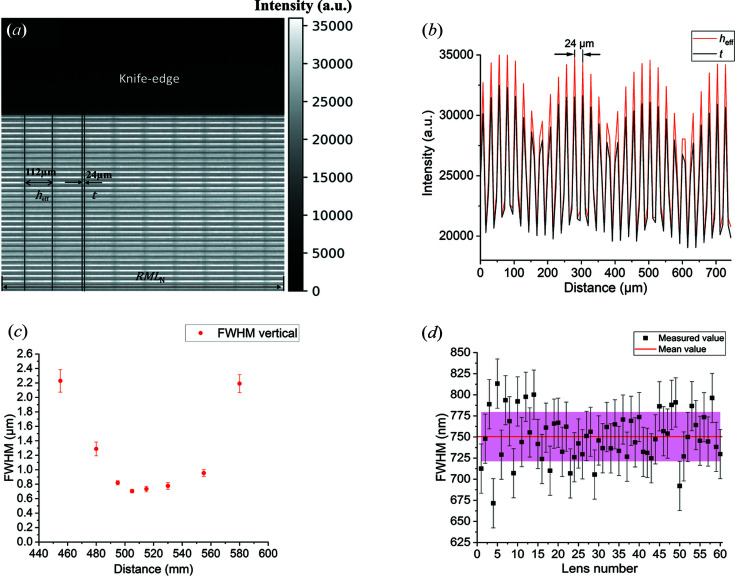
Focusing evaluation by the knife-edge scan with 100 nm steps. (*a*) One of the recorded images in the knife-edge scan; (*b*) intensity profiles through the RMLs within *h*_eff_ and transition area *t* in (*a*). The distance between intensity peaks is 24 µm. (*c*) The size of line foci (FWHM) versus the distance of the knife-edge and RML array. The focal distance is 505 mm for 35 keV X-rays. (*d*) The size of line foci (FWHM) for 60 RMLs at the focal distance. The average X-ray intensity line width is 750 ± 29 nm (vertical focus).

The experiment was performed at the medium-length bending magnet beamline BL20B2 since it provides a beam size of about 300 mm (horizontal) × 20 mm (vertical) at the specimen position. The beam flux density of 35 keV monochromatic X-rays is ∼10^8^ photons s^−1^ mm^−2^. The focal distance was measured by moving a knife-edge along the optical axis and performing a knife-edge scan at each position, searching the minimum of the focal width (FWHM). The knife-edge was moved downstream from 455 mm to 580 mm from the RML array. An sCMOS camera (Hamamatsu Photonics ORCA-Flash 4.0) coupled with a set of optics and a 200 µm-thick LuAG scintillator was used to measure intensity response through the knife-edge scan. The number of pixels of the camera was 2048 × 2048, and the effective pixel size was 8.03 µm. A slightly tapered tantalum blade was used as the knife-edge and was scanned through the foci with a step size of 100 nm. The camera was set at 50 mm downstream from the knife-edge. One of the recorded images is shown in Fig. 5 ▸(*a*), where *h*_eff_ (*h*_eff_ = *h* − *t*) and *t* were evaluated to be 112 µm and 24 µm, respectively. The intensity profiles of the regions *h*_eff_ and *t* are shown individually in Fig. 5 ▸(*b*). The intensity peaks coincide, although the intensity in the transition region *t* was slightly lower, as predicted by the ray-tracing simulations. The intervals between intensity peaks were 24 µm. The evaluated values of the focal width (FWHM) obtained by the knife-edge measurements at different distances are shown in Fig. 5 ▸(*c*). The minimum size of the X-ray line foci (FWHM) is obtained at a distance (distance between RML array and the knife-edge) of 505 mm ± 19.5 mm. Thus, the measured focal distance was 505 mm, close to the theoretical value of 520 mm. In Fig. 5 ▸(*d*) the size of line foci (FWHM) for 60 RMLs at the focal distance is presented. For each foci, the recorded intensity as a function of the knife-edge position was differentiated and fitted by the Gaussian function, resulting in an average focal size of 750 ± 29 nm (FWHM), which was consistent with the theoretical value. By substituting the value of the focal distance into equation (6), the DOF of a single lens was 32 mm, using *A*_eff_ = 24 µm. The depth of field 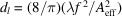 was 39 mm (Lengeler *et al.*, 1999*a* ▸). Thus, the size of line foci formed by all RMLs was almost constant in the depth of field.

## 6. Demonstration of a large FOV based on the inclined RML array   

**Figure 6:**
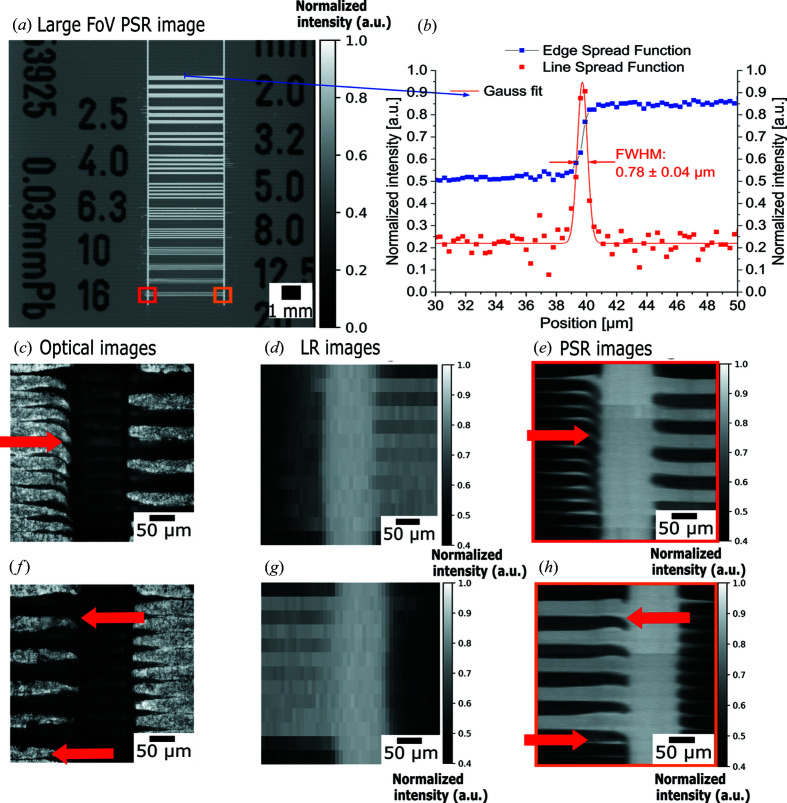
Comparison of the optical microscopy, low-resolution (LR) image (pixel size 24.09 µm × 8.03 µm) and PSR-STHXM image of the resolution test pattern: (*a*) large FOV PSR-STHXM image (pixel size 300 nm × 8.03 µm) reconstructed from 80 LR images. The scanning step of the specimen was 300 nm; the image size is 1.64 cm × 1.64 cm. (*b*) Spatial resolution evaluation by the edge spread function of the profile on the blue line in (*a*). The spatial resolution is 780 ± 40 nm. (*c*, *f*) Optical microscopy images, (*d*, *g*) LR images, and (*e*, *h*) zoomed-in views of the red and orange boxes shown in (*a*) are presented. The smallest features (25 µm line and 25 µm space) and some defects and cracks are resolved in PSR-STHXM images, as indicated by the red arrows.

For the spatial resolution evaluation in PSR-STHXM, a lead X-ray test chart (‘Type 14’, Moriyama X-ray Equipments Co. Ltd) was measured. The transmission through the test chart pattern was 50.2% at 35 keV. The geometric size of the test chart is 35 mm × 25 mm, with the smallest feature of 20 line-pairs per millimetre (25 µm lines and 25 µm space). The test chart was placed at the focal position, that is, 505 mm downstream of the inclined RML array. It was scanned with a 300 nm step size according to the Nyquist–Shannon sampling theorem (Marks, 1991 ▸), with an exposure time of 1.5 s at each step. Since the physical aperture of the inclined RML array was 24 µm, the specimen was scanned within 24 µm, resulting in 80 images. The total exposure time was 2 min, and including the dwell time (read out time of the CCD and specimen scan) of about 2 min, the total scan time was 4 min. For the proof of concept, the recorded image pixels (pixel size 8.03 µm × 8.03 µm, number of pixels 2048 × 2048) were 3 × 1 binned in the specimen scanning direction to obtain LR images with the pixel size 24.09 µm × 8.03 µm and 682 × 2048 pixels. The pixel super-resolution reconstruction (Mamyrbayev *et al.*, 2019 ▸) was performed, and the result presented in Fig. 6 ▸ demonstrates the large FOV PSR-STHXM. In Fig. 6 ▸(*a*), the PSR image of the resolution test chart pattern is presented. The PSR image size is 54560 × 2048 pixels with a pixel size of 300 nm × 8.03 µm producing an FOV of 1.64 cm × 1.64 cm. The spatial resolution was defined by the edge profile-fitting method (Ma *et al.*, 2019 ▸) from the line spread function, which is the derivative of the edge spread function (10–90% criterion was applied) shown in Fig. 6 ▸(*b*). Therefore, the spatial resolution in the scanning direction is 780 ± 40 nm, which fits with the X-ray focal width, and the spatial resolution in the perpendicular direction is 20.4 ± 1.05 µm (the spatial resolution evaluation is shown in Fig. S1 of the supporting information). For a demonstration of the advantage of the PSR technique, a comparison of the zoomed-in view [colored boxes in Fig. 6 ▸(*a*)] with the optical microscopy images [Figs. 6 ▸(*c*) and 6(*f*)] and LR images [Figs. 6 ▸(*d*) and 6(*g*)] of the same area is presented. As can be seen in Figs. 6 ▸(*e*) and 6(*h*), the smallest features of the test pattern 25 µm line and 25 µm space, defects and cracks, caused by the manufacturing process, are clearly resolved in the PSR images as well as in optical microscopy images, though are not depicted in LR images.

## 7. Application of a large FOV for studying extended and thick specimens   

**Figure 7:**
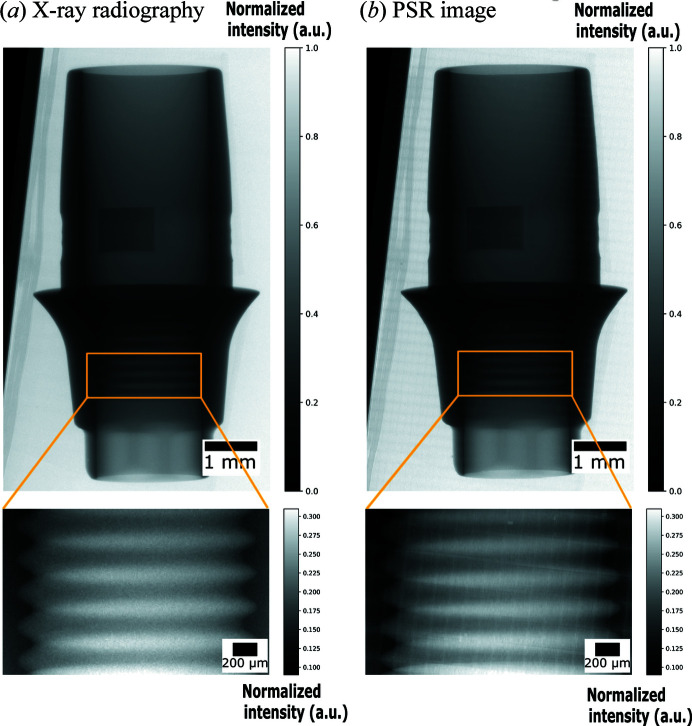
Application of the PSR-STHXM for investigating biomedical implants with a zoomed-in view of the internal thread fabricated via selective laser melting. (*a*) Conventional X-ray radiography image (pixel size 8.03 µm × 8.03 µm). (*b*) PSR-STHXM image (pixel size 300 nm × 8.03 µm).

As a possible PSR-STHXM application, a 3D printed part from a dental implant system was investigated. The so-called dental implant abutment represents the middle section of a state-of-the-art three-part dental implant system, connecting the visible prosthesis (crown) to the bone screw (implant). The abutment is fixed to the lower implant with a small screw, and the upper prothesis is cemented onto the abutment. The abutment was fabricated by selective laser melting technology from medical-grade material powder Ti6Al4V ELI (Titanium Gr. 23; Vilaro *et al.*, 2011 ▸). The dental implant system can be monitored with synchrotron-based hard X-ray radiography (Rack *et al.*, 2010 ▸). In Fig. 7 ▸, a comparison between a PSR image and conventional X-ray radiography (pixel size 8.03 µm × 8.03 µm) is performed. The scanning procedure for PSR-STHXM of the abutment was the same as that used for X-ray test chart pattern (Fig. 6 ▸). The zoomed-in view of the internal thread in the PSR image is sharper than the conventional ones.

We think that PSR-STHXM could be a useful tool for the characterization of biomedical implants. In the future, it could be possible to perform computed tomography studies of these specimens in three dimensions, allowing us to investigate in detail the surface roughness, deviation from the CAD-design, as well as cracks and defects caused by the selective laser melting fabrication technique.

## 8. Discussion   

In the present work, the inclined RML array was fabricated on a 525 µm silicon substrate. As the substrate was tilted by α = 30°, the absorbing length of the substrate was doubled (*i.e.* 1050 µm), increasing the absorption of X-rays by the substrate to 21% at 35 keV. Therefore, in the future, the substrate material should be changed to Vespel (DuPont), peek, or even pure 2 µm titanium membrane for less absorption by the substrate and to increase the intensity of foci. Furthermore, the fabricated RMLs present the same geometry, which leads to different focal distances in a staircase arrangement; the lens geometry from RML_1_ to RML_N_ could be designed in such a way to obtain an identical focal position for all RMLs by changing the radius of curvature accordingly.

It should be emphasized that the array of straight RMLs oriented perpendicularly to the substrate, *i.e.* without inclination, can also be utilized to enlarge the FOV by tilting the substrate. However, under X-ray illumination, the straight RMLs will generate the tilted X-ray line foci corresponding to the substrate tilt angle. Moreover, the Scheimpflug effect is much more extensive for straight structures than for the proposed staircase array of inclined RMLs, if the FOV in the centimetre range is desired.

The inclined RML array could be used as a hard X-ray multi-lens interferometer (Snigirev *et al.*, 2014 ▸; Lyubomirskiy *et al.*, 2016 ▸), since X-rays from line foci diverge, overlap in the far field and create an interference pattern. Also, in the future, the inclined RML array can also be used for non-interferometric multimodal X-ray imaging since it provides an array of X-ray foci (dos Santos Rolo *et al.*, 2018 ▸; Reich *et al.*, 2018 ▸).

Two-dimensional PSR-STHXM (Mamyrbayev *et al.*, 2020 ▸) would be feasible by generating an array of point foci with a couple of inclined RML arrays aligned orthogonally, providing an FOV in the centimetre range. Raster-scanning or ‘on-the-fly’ scanning of the specimen through the point foci would allow us to perform PSR-STHXM in two dimensions.

## 9. Conclusions   

In this work, a new optical component, staircase array of inclined RMLs fabricated by tilted exposure deep X-ray lithography and electroplating, was presented. Ray-tracing simulations demonstrated the proof of concept successfully, and the performance of the inclined RML array was measured experimentally at the BL20B2, SPring-8 using 35 keV X-rays, resulting in an FOV of about 5 cm (24 µm aperture × 2000 lenses) × 2.5 cm (136 ± 4 µm lens height × 189 lenses).

The resultant spatial resolution (780 ± 40 nm) of PSR-STHXM based on the inclined RML array was comparable with the size of line foci in the scanning direction. In the direction perpendicular to the scanning direction, it was equal to 20.4 ± 1.05 µm.

As a possible application of the developed technique, we demonstrated PSR-STHXM of a dental implant abutment made of titanium alloy. As a next step, the development of three-dimensional PSR-STHXM tomography would allow the identification of the shape of additively manufactured parts and could reveal possible cracks or defects inside the parts. This information is of high interest for an industrial manufacturer or in material sciences to assure production quality and mechanical stability, especially of filigree thin-walled medical implants typically present in dental implantology. Other possible applications of the inclined RML array involve the development of single-shot multimodal X-ray imaging and as a hard X-ray multi-lens interferometer. Therefore, the inclined RML array is a promising optics for synchrotron-based X-ray applications.

## Supplementary Material

Spatial resolution evaluation in the direction perpendicular to the scanning direction. DOI: 10.1107/S1600577521001521/yi5100sup1.pdf

## Figures and Tables

**Table 1:** Main parameters of the fabricated inclined RML array

	α + β (°)	*h* (µm)	*N*_v_	*N*	*d* (µm)	*w* (µm)	*L*_d_ (µm)	*A*_ph_ (µm)	*R* (µm)
Design value (at α = 30°)	90	132	2000	189	130	3.5	99	24	1.5
Measured value (at α = 0°)	59.8 ± 0.2	118 ± 4.2	2000	189	150 ± 0.2	4.56	110	24	1.4
Calculated value (at α = 30°)	90	136 ± 4.2	2000	189	129.9 ± 0.2	3.95	95.3	24	1.57
