# Supplementary material for: Staircase array of inclined refractive multi-lenses for large field of view pixel super-resolution scanning transmission hard X-ray microscopy
Source: J Synchrotron Radiat. 2021 Mar 12;28(Pt 3):732–40. doi: 10.1107/S1600577521001521 (PMC8127365; doi:10.1107/S1600577521001521)
Supplement: Supplementary file 1 [file s-28-00732-sup1.pdf]

JOURNAL OF SYNCHROTRON RADIATION

Volume 28 (2021)

Supporting information for article:

Staircase array of inclined refractive multi-lenses for large field of view pixel super-resolution scanning transmission hard X-ray microscopy

Talgat Mamyrbayev, Katsumasa Ikematsu, Hidekazu Takano, Yanlin Wu, Kenji Kimura, Patrick Doll, Arndt Last, Atsushi Momose and Pascal Meyer

S1. The spatial resolution evaluation in the direction perpendicular to the scanning direction.

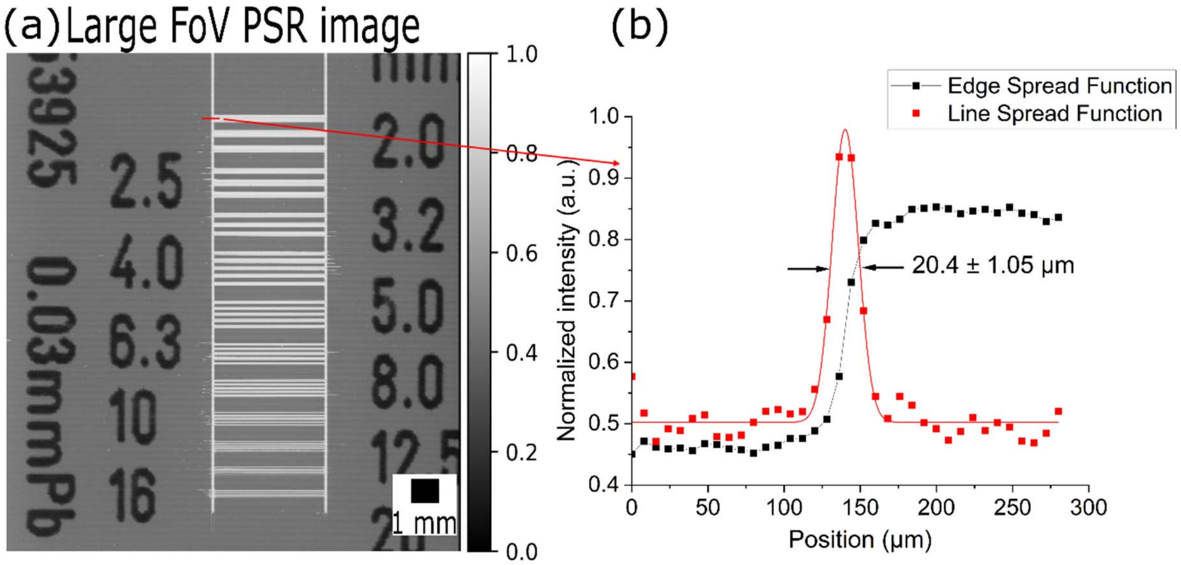

Figure S1 (a) Large FoV PSR-STHXM image (pixel size 300 nm × 8.03 µm). (b) Spatial resolution evaluation by the edge spread function of the profile on the red line in (a). The spatial resolution is 20.4 ± 1.05 µm (FWHM).
